# Jabuticaba [*Plinia trunciflora* (O. Berg) Kausel] Protects Liver of Diabetic Rats Against Mitochondrial Dysfunction and Oxidative Stress Through the Modulation of SIRT3 Expression

**DOI:** 10.3389/fphys.2021.665747

**Published:** 2021-07-06

**Authors:** Caroline Calloni, Luana Soares Martínez, Daniela Franciele Gil, Douglas Machado da Silva, Matheus Parmegiani Jahn, Mirian Salvador

**Affiliations:** ^1^Laboratório de Estresse Oxidativo e Antioxidantes, Instituto de Biotecnologia, Universidade de Caxias do Sul (UCS), Caxias do Sul, Brazil; ^2^Laboratório de Fisiologia e Farmacologia, Universidade de Caxias do Sul (UCS), Caxias do Sul, Brazil

**Keywords:** diabetes, sirtuins, phenolic compounds, electron transport chain, mitochondria, jaboticaba

## Abstract

Complications generated by hyperglycemia present in diabetes mellitus (DM) have been constantly related to oxidative stress and dysfunction in the mitochondrial electron transport chain (ETC). Sirtuin 3 (SIRT3), which is present in mitochondria, is responsible for regulating several proteins involved in metabolic homeostasis and oxidative stress. Studies have suggested alterations in the expression of SIRT3 in DM. The objective of this study was to evaluate the effects of phenolic compounds in jabuticaba (*Plinia trunciflora*), a berry native to Brazil, on the activity of mitochondrial ETC complexes, SIRT3 protein expression, and oxidative stress parameters in liver of diabetic rats induced by streptozotocin. After type 1 DM induction (streptozotocin 65 mg/kg), diabetic and healthy rats were treated with jabuticaba peel extract (JPE) by gavage (0.5 g/kg of weight) for 30 days. After treatments, those diabetic rats presented impaired activities of complexes I, II, and III of ETC along with an overexpression of SIRT3. In addition, an increase in lipid peroxidation and superoxide dismutase and catalase activities was observed in the diabetic group. The treatment with JPE was able to recover the activity of the mitochondrial complexes and reduce the expression of SIRT3. Furthermore, JPE treatment reduced oxidative damage to lipids and brought the antioxidants enzyme activities to basal levels in diabetic rats. Together, these results demonstrate that JPE can reduce oxidative stress related to DM by restoring mitochondrial complexes activity and regulating SIRT3 expression. Thus, JPE could become an alternative to reduce the development of complications related to DM.

## Introduction

Diabetes mellitus (DM) is a metabolic disease characterized primarily by hyperglycemia, as a result of the absolute absence of insulin, resistance to the action of insulin, or both. Type 1 DM (DM1) is characterized by an absolute insulin deficiency, usually due to an autoimmune destruction of pancreatic beta cells (American Diabete Association, 2014). This type constitutes about 5–10% of all cases of DM. A meta-analysis conducted by Mobasseri et al. (2020), through the analysis of 193 studies between 1999 and 2019, demonstrated an incidence of 15 cases for every 100,000 individuals and a prevalence of 9.5 cases for every 10,000 individuals in the world. Although DM1 can be treated with the use of insulin and despite recent technological advances to facilitate the treatment, data show that less than 30% of patients with DM1 are able to achieve adequate glycemic control (glycated hemoglobin less than 7.5%), and the disease is associated with a high risk of cardiovascular disease, a higher risk of end-stage renal disease, when compared with patients with DM2, and premature mortality (Secrest et al., 2010; Baxter et al., 2016; Iqbal et al., 2018).

Among the main DM-related alterations, oxidative stress has been widely reported and is presumed to occur as a result of mitochondrial dysfunction (Thapa et al., 2010; Semaming et al., 2014; Cieluch et al., 2020; Sobhi, 2020). Mitochondrial complexes I and II have increased activity and complex III (CIII) presents reduced activity, both in insulin deficiency and in insulin resistance states (Raza et al., 2011; Zhang et al., 2011; Blake and Trounce, 2013). An impairment in the activity of mitochondrial complexes can compromise the transfer of electrons between these complexes in the electron transport chain (ETC) which, in turn, can lead to the leakage of these electrons and, ultimately, may favor the increased generation of reactive oxygen species (ROS) (Brownlee, 2001; Blake and Trounce, 2013; Quinlan et al., 2013; Tiwari et al., 2013). Increased ROS can interact with lipids, proteins, and nucleic acids, leading to loss membrane integrity; structural or functional changes in proteins; and genetic mutations (Pisoschi and Pop, 2015). Studies have shown the involvement of oxidative stress and dysfunction of the activity of mitochondrial complexes in complications related to DM1, such as cardiomyopathy and liver disease (Regnell and Lernmark, 2011; Al-Hussaini et al., 2012; Mohamed et al., 2016; Rukavina-Mikusic et al., 2021).

Sirtuins (silent information regulators or SIRT) are a family of NAD^+^-dependent deacetylase proteins, distributed in various cell compartments, and exhibit a variety of functions (Li et al., 2015). In mammals, seven different sirtuins (SIRT1-7) are known. SIRT3, one of the sirtuins found in mitochondrial, regulates many of the mitochondrial proteins involved with metabolic homeostasis, oxidative stress, and cell survival by reversible enzyme deacetylation (Hirschey et al., 2011). In general, enzymatic activity of SIRT3 substrate proteins increases following deacetylation, suggesting that protein acetylation suppresses mitochondrial function (Anderson and Hirschey, 2012). SIRT3 is highly expressed in the brain, heart, kidney, brown adipose tissue, and liver (Nogueiras et al., 2012). The function of SIRT3 on DM1 complication is not completely clear; therefore, more studies are needed to understand the influence of SIRT3 expression on DM1.

The search for affordable natural products for the treatment of DM and its complications has increased significantly in recent years, since conventional DM treatment is expensive and may not always be effective (Newman and Cragg, 2016), mainly to reduce oxidative stress and mitochondrial dysfunction. In this context, the phenolic compounds have a prominent place, as several studies have associated the pathogenesis of DM with oxidative stress (Maiese, 2015; Ullah et al., 2016). Phenolic compounds comprise a group of substances widely distributed in plants and have one or more hydroxyl groups attached to aromatic rings (Lin et al., 2016). Several studies have demonstrated the biological activities of these compounds, among them, antioxidant, anti-inflammatory (Bak et al., 2013; Rocha et al., 2015), antitumor (Duo et al., 2012; Fantini et al., 2015), and antimicrobial activities (Tohma et al., 2016; de Camargo et al., 2017). Moreover, these compounds can act as signals influencing the expression of several proteins, such as SIRT3 (Cerbaro et al., 2020; Rigotti et al., 2020), resulting in modulation of the cellular response in the various conditions (Kim et al., 2014).

Jabuticaba (*Myrtaceae*) is a fruit native to Brazil that has a globular shape, with a thick purple peel, and a white gelatinous pulp, and bittersweet flavor (Lorenzi, 2011). In Brazil, jabuticaba trees are distributed in the center, south, and southeast of the country, with secondary dispersion points in Paraguay and Argentina (Citadin et al., 2007). In these areas, jabuticaba fruit is part of the population’s diet, and in 2006, 3,052 tons of fruit were produced in Brazil (IBGE, 2006). After harvesting jabuticaba is very perishable, with a useful life of about 3 days, probably due to the high content of water and sugars. As a result, the fruit is widely used in the manufacture of jellies, juices, wines, liqueurs, and vinegars (Brunini et al., 2004; Balerdi et al., 2006). Studies have shown that the three main species of jabuticabas naturally distributed in Brazil (*Plinia jaboticaba, Plinia caulifora, and Plinia trunciflora*) are rich in phenolic compounds, such as anthocyanins and flavonoids, concentrated mainly in the peel (Wu et al., 2012; Calloni et al., 2015; Neves et al., 2018). However, few studies have focused on the investigation of phenolic compounds of *P. trunciflora* and their biological activities related to DM, and the existing studies focus on DM2 models and used another species of jaboticaba (*M. jaboticaba*) (Lenquiste et al., 2012; Dragano et al., 2013; Plaza et al., 2016). Despite the observed evidence, there are no studies in the literature that have investigated the effects of the aqueous extract of *P. trunciflora* peel in a DM1 model. Thus, this study used a rat model that had DM1 induced by a single dose of streptozotocin to study the effects of *P. trunciflora* peel aqueous extract on DM1. Therefore, the objective of this study was to evaluate the effects of *P. trunciflora* peel aqueous extract on the function of the ETC complexes, the expression of SIRT3, and parameters of oxidative stress in the liver of streptozotocin-induced diabetic rats.

## Materials and Methods

### Chemicals

Antibodies were purchased from Santa Cruz Biotechnology Inc. (Dallas, TX, United States). Sucrose was purchased from Vetec Quimica Fina Ltda. (Rio de Janeiro, Brazil). Streptozotocin, sodium citrate, citric acid, Tris, EDTA, albumin, potassium cyanide, NADH, ubiquinone, rotenone, succinate, 2,6-dichlorophenolindofenol, decylubiquinone, ubiquinol, cytochrome c, antimycin A, adrenaline, glycine, hydrogen peroxide, malondialdehyde, butanol, and tween-20 were obtained from Sigma (St. Louis, MO, United States). All the chemicals were of analytical grade.

### Plant Material and Sample Preparation

The collection of the jabuticabas was carried out in the cities of Caxias do Sul (29°10'55.31'S; 51°12'9.70'O) and Veranópolis (28°56'26.00'S; 51°32'35.1'O), Grande do Sul, Brazil, in 2017. Identification of the voucher specimens was performed by the herbarium of the University of Caxias do Sul, RS (HUCS40706). An access authorization (A1009E) was obtained from the Sistema Nacional de Gestão do Patrimônio Genético (SISGEN), and collection authorization (no. 38096-3) was requested from the Sistema de Autorização e Informação em Biodiversidade (SISBIO). For extract preparation, first jabuticaba peels were removed manually and ground in a Wiley mill. The extraction was performed with distilled water (5% w/v) under reflux (100°C) for 15 min. Afterward, JPE was lyophilized (LIOBRAS model L-101) and stored at −80°C until the tests (Calloni et al., 2015). Our previously published study demonstrated that JPE is rich in phenolic compounds, and these compounds, determined through the full mass spectrum analysis, include myricitrin, pedunculagin, kaempferol, cyanidine-3-O-glycoside, and ellagic acid (Calloni et al., 2020).

### Animals

Twenty-four male Wistar rats, weighing about 355.85 ± 36 g and 90 days old, were obtained from the Center for Reproduction and Experimentation of Laboratory Animals of the Federal University of Rio Grande do Sul. The animals were housed in plastic cages (three animals in each) and received water and pelleted food *ad libitum.* They were maintained under controlled temperature (23 ± 2°C) and 12-h light–dark cycle in the Laboratory of Physiology and Pharmacology at the University of Caxias do Sul. All the procedures performed were in accordance with the National Council for Control of Animal Experimentation (CONCEA) and Brazilian Society of Laboratory Animal Science (SBCAL), and the experimental protocol was approved by the Ethics Committee for Animal Research of the University of Caxias do Sul (001/2017).

### Diabetes Induction

Intraperitoneal injection of streptozotocin (65 mg/kg solution – STZ, Sigma Chemical Company), diluted in sodium citrate buffer (0.01 mol/l pH 4.5) and citric acid (0.01 mol/l pH 4.5), was used to induce DM1 (Furman, 2015; Wu and Yan, 2015; Rech et al., 2020). The animals were in fasting for the administration of streptozotocin. Healthy animals received the same volume of buffer (2 ml/kg). Blood glucose levels were measured through a glycosometer (ACCU-CHEK) after 72 h, and the animals that presented glycemia greater than 200 mg/dl were considered diabetic (Al-Malki and El Rabey, 2015; Radenković et al., 2016).

### Experimental Design and Samples

For the extract treatment, the animals were randomly divided into four groups containing six animals each. The extract dissolved in water was administered once a day by gavage for 30 days, always at 8 a.m. The concentration of JPE of 0.5 g/kg was defined based on the concentrations of jabuticaba extract used in the study by Alezandro et al. (2013). Animals were weighed every week, and the dose (0.5 g/kg) of extract was adjusted considering the current weight of each animal. The groups were organized as follows: *Healthy group* (*n* = 6): animals receiving only water; *JPE group* (*n* = 6): healthy animals receiving extract dissolved in water (0.5 g/kg); *DM group* (*n* = 5): diabetic animals receiving water; and *DM + JPE group* (*n* = 6): diabetic animals receiving extract dissolved in water (0.5 g/kg).

After 30 days of treatment, the animals were euthanized through decapitation. At the time of euthanasia, the animals were fasting for 4 h. Livers were removed, rinsed of any adhered blood using saline buffer, and frozen at −80°C. Prior to analysis, livers were quickly sliced, and fragments were homogenized in appropriate buffers.

### Tissue Homogenization

To evaluate the complexes of the ETC activity, mitochondria were extracted from tissue. The separation of mitochondria was performed as described by Frezza et al. (2007) with some modifications. The liver was homogenized in ice cold buffer containing 0.1 M of Tris–MOPS, 0.1 M of EDTA/Tris, and 1 M sucrose, pH 7.4. Subsequently, the samples were centrifuged at 600 × *g* for 10 min at 4°C. The supernatant was collected and centrifuged again at 7,000 × *g* for 10 min at 4°C. The resulting supernatant was discarded, and the pellet washed with 200 μl of ice-cold sucrose buffer, followed by further centrifugation at 7,000 × *g*, and the resulting supernatant was again discarded. The pellet was used to determine total proteins, by the method of Bradford (1976), and activity of the complexes I–IV of the ETC.

To evaluate the activity of the enzymes superoxide dismutase (SOD) and catalase (CAT), oxidative damage to lipids, and the expression of SIRT3, the tissue was homogenized in a Potter-Elvehjem pestle and glass tube using ice cold phosphate buffered saline (PBS). Then, the homogenate was centrifuged at 1,000 × *g* for 10 min, at 4°C.

### Activity of the Mitochondrial ETC

The function of the complexes I–IV of ETC was evaluated by spectrophotometric methodology described by Spinazzi et al. (2012). For this assay, the isolated mitochondria were mixed with a potassium phosphate buffer (0.05 M, pH: 7.5) containing bovine albumin (3 mg/ml) and potassium cyanide (KCN, 300 μm). The NADH substrate (100 μm), electron acceptor ubiquinone (60 μm), and specific rotenone inhibitor (10 μm) were added to evaluate the activity of complex I (CI) of the ETC. The activity of complex II (CII) was evaluated with the addition of substrate succinate (20 mm), 2,6-dichlorophenolindofenol (80 μm), electron acceptor decylubiquinone (DUB, 50 μm), and 2-thenoyltrifluoroacetone (10 μm). The substrate ubiquinol (DubH2, 100 μm), cytochrome c (75 μm as electron acceptor, and antimycin A (10 μg/ml) as a specific inhibitor were used to determine the activity of CIII. To evaluate complex IV (CIV), reduced cytochrome c was used as the substrate and KCN (300 μm) was used as a specific inhibitor. The kinetics was made spectrophotometrically at wavelengths specific to each complex. Results are expressed as nmol/min/mg of protein.

### Immunoblotting Assay

To determine the possible mechanism by which the extract modulates the activity of the ETC complexes, the expression of the SIRT3 protein was determined. For this, tissue homogenized in PBS was used. Proteins were denatured and separated on 12% SDS-PAGE gel. After separation, the proteins were transferred to an Immobilon-P (Millipore^®^) transfer membrane. Subsequently, the membranes were blocked with 5% non-fat milk and incubated for 2 h with the primary antibody. Sirtuin 3 (SIRT3-28 kDa; 1:1,000) was used as the primary antibody. β-tubulin (55 kDa) was used as constitutive protein. After incubation with the primary antibody, the membranes were repeatedly washed with PBS-t (PBS-buffered saline containing 0.1% Tween-20) to remove unbound primary antibody. The membranes were then stained with anti-rabbit IgG conjugated–peroxidase antibodies (1:2,000) for 1 h at room temperature. Membranes were washed repeatedly. Protein detection was performed by using a chemiluminescence protocol (Amersham Bioscience). Protein band images were captured using ImageQuant (LAS 500 – GE Healthcare), and pairwise comparisons of the protein bands on the immunoblot were performed using ImageJ 1.45 software.

### Antioxidant Enzymes

The activity of the antioxidant enzymes SOD and CAT was determined in homogenized tissue. SOD activity was determined by measuring the inhibition of self-catalytic adrenochrome formation rate at 480 nm, in a reaction medium containing 1 mmol/l adrenaline (pH 2.0) and 50 mmol/l glycine (pH 10.2). This reaction was performed at 30°C for 3 min (Bannister and Calabrese, 1987). Results are expressed as USOD (units of SOD)/mg of protein. One unit is defined as the amount of enzyme that inhibits the rate of adrenochrome formation by 50%. CAT activity was measured according to the methods described by Aebi (1984). The assay measures the decomposition rate of H_2_O_2_ at 240 nm. The reaction was conducted at 30°C for 1 min. Results are expressed as mM H_2_O_2_/min/mg of protein. All absorbances were measured in spectrophotometer (SHIMADZU, model UV-1700).

### Lipid Peroxidation

Lipid peroxidation (LPO) was evaluated through the measurement of thiobarbituric acid reactive substances, which is based on the colorimetric reaction of malondialdehyde (MDA), one of the final products of LPO. Briefly, proteins were precipitated using trichloroacetic acid (5%). After centrifugation, the pellet was dissolved in sulfuric acid (3 M) and color reagent (containing 0.03 M thiobarbituric acid, 0.1 M sodium bicarbonate, and 1.5 M sodium sulfate) and heated to 100°C for 15 min. Afterward, 1.75 ml of butanol was added and centrifuged. The supernatant readout was made spectrometrically at 530 nm (Wills, 1966). Results are expressed as μm of MDA/mg of protein.

### Statistical Analysis

The results were expressed as the mean ± standard error (SE). The data were determined to be parametrical or non-parametrical by using the Shapiro–Wilk test. Data were submitted to two-way ANOVA. A statistical significance of *p* < 0.05 was considered. The software SPSS 22.0 (SPSS Inc., Chicago, IL, United States) was used for all the statistical analysis.

## Results

As expected, both groups of rats that received intraperitoneal injection of streptozotocin showed increased blood glucose levels (624.00 ± 26.02 mg/dl and 629.20 ± 23.62 mg/dl), compatible with DM. The group of diabetic rats that was treated with JPE maintained high blood glucose levels demonstrating that the extract had no effect on this parameter. On the other hand, rats that did not receive streptozotocin had normal blood glucose levels (125.20 ± 4.76 mg/dl). The treatment of healthy rats with JPE did not affect blood glucose levels, which remained into normal levels (121.40 ± 2.94 mg/dl; Calloni et al., 2020).

**Figure 1:**
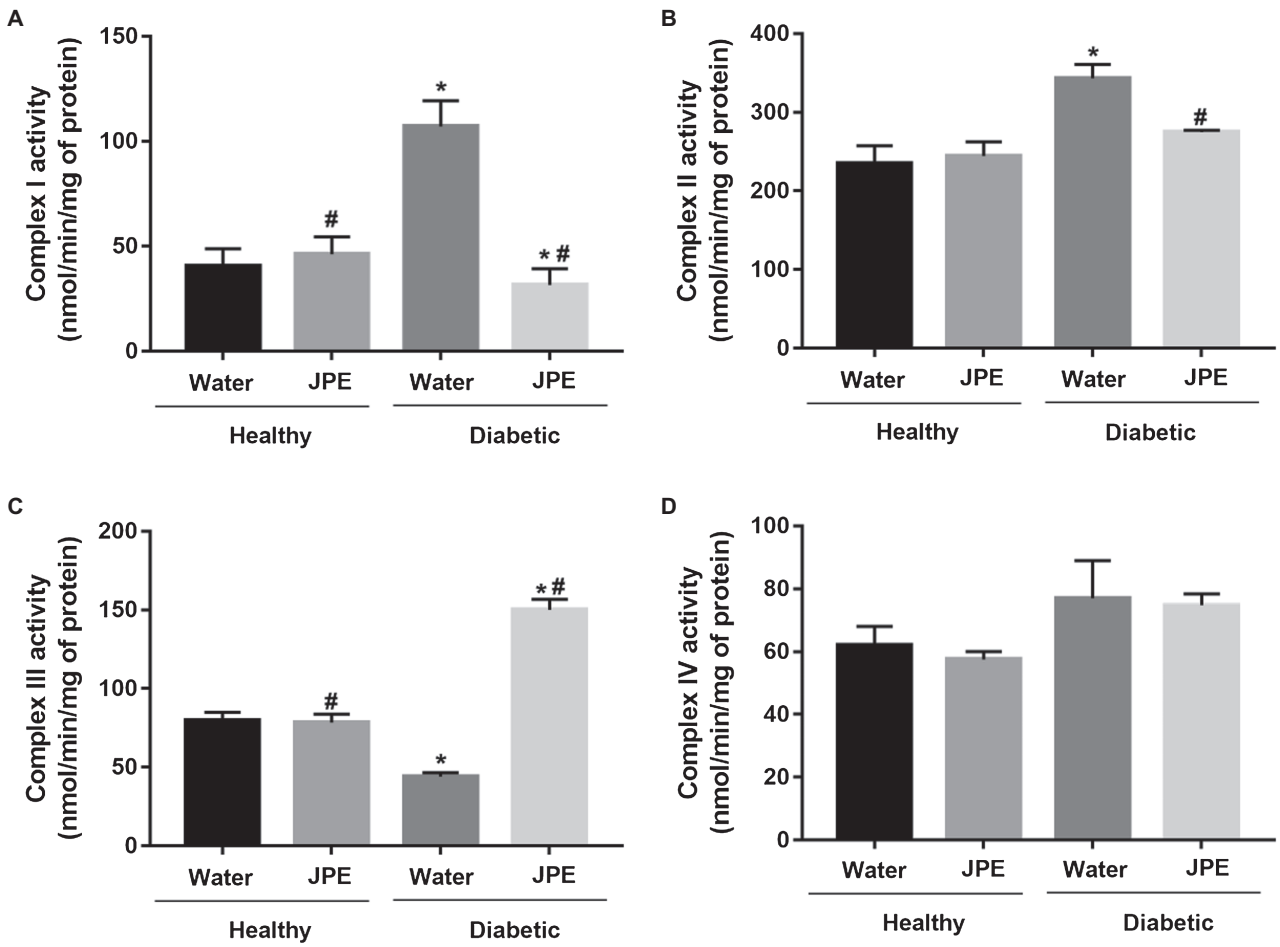
Activity of complexes I **(A)**, II **(B)**, III **(C)**, and IV **(D)** of the liver mitochondrial electron transport chain of healthy (control group) and diabetic rats (DM) treated with or without 0.5 g/kg jabuticaba peel extract (JPE). Data are presented as mean and standard error (SE; *n* = 5 to 6 rats/group). ^*^Indicates statistically significant difference in relation to healthy groups. ^#^Indicates statistically significant difference in relation to groups without JPE treatment. Statistical significance of *p* < 0.05.

The activity of CI to IV of the mitochondrial ETC of liver from healthy and diabetic rats, treated with or without JPE, was evaluated. The activity of CI and CII was significantly increased in the DM group compared to the control group (Figures 1A,B). On the other hand, the activity of CIII was significantly decreased in the DM group, demonstrating a mismatch between the activities of the complexes during DM (Figure 1C). After the 30-day treatment with JPE, the activities of the mitochondrial complexes exhibited modifications, with significantly decreased activity of the CI and CII and increased activity of the CIII (Figures 1A–C). There was no change in the activity of the CIV (Figure 1D). In addition, no change in the activity of the complexes was observed in healthy rats that received the JPE. The two-way ANOVA analysis demonstrated a statistically significant interaction between the effect of treatment with JPE and the presence or absence of DM in CI, CII, and CIII activity (*p* = 0.0001, *p* = 0.031, *p* = 0.0001, respectively). This means that JPE modulates CI, CII, and CIII activity only when DM is present. The interaction between JPE treatment and the presence or absence of DM on CIV activity was not statistically significant.

**Figure 2:**
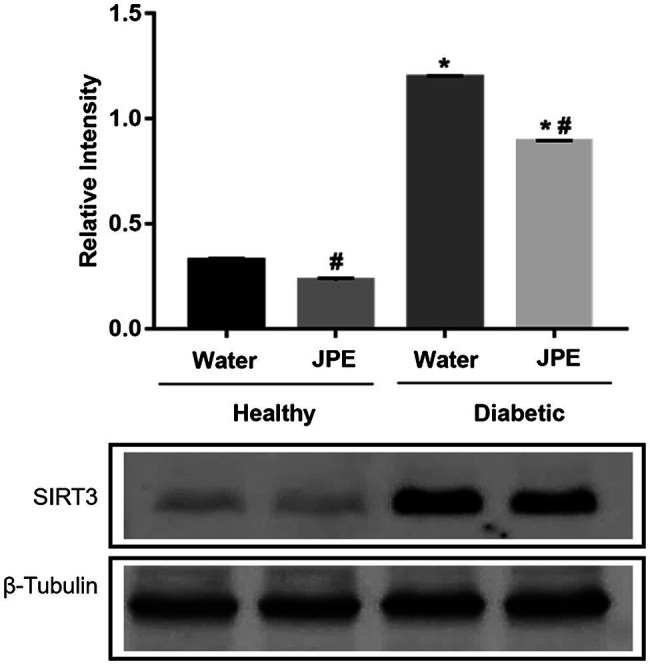
Expression of SIRT3 in liver of healthy (control group) or diabetic (DM) rats treated with or without 0.5 g/kg of JPE. Data are presented as mean and standard error (SE) of at least three replicates. *Indicates statistically significant difference in relation to healthy groups. ^#^Indicates statistically significant difference in relation to groups without JPE treatment. Statistical significance of *p* < 0.05.

Regarding the expression of SIRT3, it was observed that the animals of the DM group displayed an overexpression of SIRT3 compared to the control group. The supplementation of JPE in diabetic rats significantly decreased the overexpression of SIRT3 (Figure 2). There was a statistically significant effect of the interaction between the treatment with JPE and the presence or absence of DM in SIRT3 expression (*p* = 0.0001).

**Figure 3:**
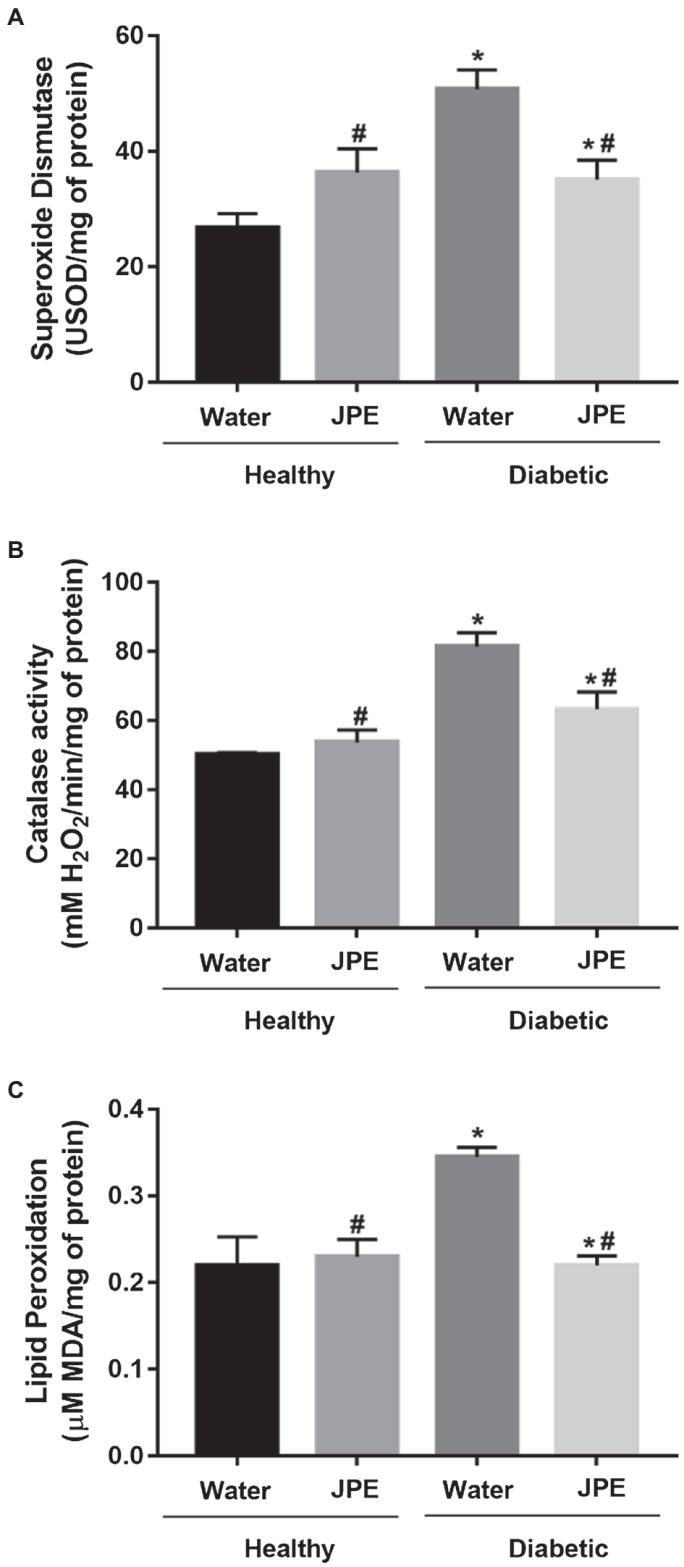
Superoxide dismutase **(A)** and catalase **(B)** activity and lipid peroxidation levels **(C)** in liver of healthy (control group) or diabetic (DM) rats treated with or without 0.5 g/kg of JPE. Data are presented as mean and standard error (SE; *n* = 5–6 rats/group). ^*^Indicates statistically significant difference in relation to healthy groups. ^#^Indicates statistically significant difference in relation to groups without JPE treatment. Statistical significance of *p* < 0.05.

Changes in the activity of the ETC complexes may lead to increased production of ROS and, in turn, could lead to the development of oxidative damage to biomolecules and an increase in oxidative stress. Therefore, the generation of ROS was evaluated indirectly by determining the activity of the antioxidant enzymes, SOD and CAT. An increase in the activity of SOD and CAT enzymes was observed in rats of the DM group (Figures 3A,B). Even with increased activity of antioxidant enzymes, LPO increased in the DM group (Figure 3C), demonstrating that the compensatory increase in antioxidant defenses is not enough to prevent oxidative damage in diabetic rats. On the other hand, when diabetic rats received JPE supplementation, the activity of SOD and CAT enzymes presented values near the basal level concomitantly with a decrease in oxidative damage to lipids (Figures 3A–C). In addition, the interaction between treatment with JPE and the presence or absence of DM had a statistically significant effect on the activity of antioxidant enzymes (SOD *p* = 0.002 and CAT *p* = 0.044) and lipid peroxidation (*p* = 0.007), demonstrating that JPE could only reduce these parameters when there are alterations induced by DM.

## Discussion

Some studies have demonstrated the presence of mitochondrial dysfunction during DM, which can, in turn, lead to oxidative stress (Sivitz and Yorek, 2010; Blake and Trounce, 2013; Wada and Nakatsuka, 2016). Evidence suggests that the mitochondrial ETC function is regulated by the SIRT3 protein (Bause and Haigis, 2013; Parodi-Rullán et al., 2018). Investigations have found changes in SIRT3 expression in DM; however, this evidence remains controversial (Bagul et al., 2015; Yu et al., 2017; Li et al., 2018). Studies have also shown that phenolic compounds have the potential to regulate the expression of several proteins, including sirtuins, and thus regulate mitochondrial function and oxidative stress (Cerbaro et al., 2020; Sun et al., 2020). Therefore, these compounds could be allies in the prevention of complications related to DM.

Jabuticaba (*P. trunciflora*) is a fruit rich in phenolic compounds, and some studies have already demonstrated its antioxidant potential (Calloni et al., 2015; Sacchet et al., 2015). Nevertheless, its effect on complexes of mitochondrial ETC activity and SIRT3 expression in diabetic rats had not been studied. This study verified mitochondrial dysfunction in diabetic animals, with an increase in the activity of CI and CII of the ETC, which may be due to the increase in the influx of electrons in these two complexes. On the contrary, a decrease in the activity of CIII was observed. The activity of CIV did not present alteration in the diabetic group. Other studies have also found dysfunction of the ETC of diabetic rats (Raza et al., 2011) demonstrated a significant increase in CI and CII activity and a reduction in CIII and CIV activity in the liver of diabetic rats induced by streptozotocin. Zhang et al. (2011) observed a significant reduction in CIII activity of the ETC in the kidney of diabetic mice, accompanied by an overactivity of CI. Alimujiang et al. (2020) also demonstrated increased CI and CII activity of the ETC in the liver of streptozotocin-induced diabetic rats.

The diabetic rats who received the JPE exhibited modulated activity of the ETC complexes, with reduced activity of the CI and CII, with the values returning to the basal levels. For the CIII, the treatment with JPE induced a significant increase in the activity, showing that the JPE can modulate the ETC activity. Corroborating our study, Gambato et al. (2018) demonstrated an increase in CI activity in hyperglycemic endothelial cells, and the treatment with *Pleurotus albidus* extract, rich in phenolic compounds, was able to bring CI activity to the basal levels. Mustata et al. (2005) also demonstrated that streptozotocin-induced diabetic rats showed a decrease in the specific activity of CIII in renal cortex and suggested that glycation may damage the CIII proteins. In addition, studies have shown that aldehydes derived from lipid peroxidation may lead to inhibition of CIII (Picklo et al., 1999; Musatov and Robinson, 2012). We also observed increased lipid peroxidation in diabetic rats, which could have led to inhibition of CIII. To understand how the regulation of changes in mitochondrial function observed in DM occurs, this study evaluated SIRT3 expression. SIRT3, a mitochondrial sirtuin, is responsible for regulating several proteins through deacetylation, such as ETC proteins and enzymes, including SOD, and thus regulates the metabolic activity and oxidative stress of this organelle (Bause and Haigis, 2013; Parodi-Rullán et al., 2018). We observed an overexpression of SIRT3 in the diabetic group. A study conducted in culture of hepatocyte cells (HepG2) and embryonic kidney cells (HEK293) demonstrated that hyperglycemia induces SIRT3 overexpression (Gounden et al., 2015). Li et al. (2018) also found an overexpression of SIRT3 in hepatocytes of high-fat-diet-induced diabetic rats. Other authors have found that SIRT3 expression is down-regulated in DM1. Yu et al. (2017) showed that SIRT3 expression was decreased in myocardial of streptozotocin-induced diabetic rats. In addition, Bagul et al. (2015) demonstrated that the expression of SIRT3 in the cardiac tissue of DM1 rats decreased. The results on the expression of SIRT3 in DM are still controversial. However, the overexpression of SIRT3 observed in our study may explain the increased activity of ETC CI and CII (Bause and Haigis, 2013; Parodi-Rullán et al., 2018). Whereas SIRT3 is responsible for regulating the activity of ETC complexes and antioxidant enzymes, the overexpression of SIRT3 could be interpreted, in the context of this study, as a compensatory response to metabolic stress induced by DM, since overexpression of SIRT3 seems to be protective, as demonstrated in several studies (Liu et al., 2017; Mao et al., 2020).

The cause of increased SIRT3 expression in diabetic rats remains unclear. However, we hypothesized that even with the increased activity of ETC CI and CII, the decrease in CIII activity would lead to a decrease in ATP generation, which in turn, could be signaling the overexpression of SIRT3, as a compensatory response to induce greater energy production. Vassilopoulos et al. (2014) found that SIRT3 is sensitive to intracellular ATP levels and regulates the ATP synthase. In addition, a study by Wu et al. (2017) with streptozotocin-induced diabetic rats demonstrated a decrease in intracellular ATP levels, even with the presence of increased activity in the ETC complexes in the pancreas. Furthermore, Rato et al. (2014) showed an increase in the activity of CI concomitant to a decrease in mitochondrial CIII activity, in which this dissociation of activities of the complexes led to the impairment of ATP formation in testicles of pre-diabetic rats. Future studies with ATP cell quantification would be important to better elucidate this regulation.

After treatment with JPE, a significant decrease in SIRT3 expression was observed. How the JPE regulates the expression of this protein is not fully elucidated; however, we hypothesize that as treatment with JPE leads to normalization of the activity of mitochondrial ETC complexes and, consequently, normalization of ATP and ROS levels, stimuli for SIRT3 expression decrease, since SIRT3 is a central component in modulating antioxidant defense and conferring resistance to oxidative stress-induced damage under hyperglycemic condition (Gounden et al., 2015).

The alteration in the function of mitochondrial complexes could explain the increase of oxidative stress during DM. This study also observed an increase in oxidative damage to lipids in the DM group, which may be a consequence of an overproduction of superoxide radical ($O_{2}^{•-}$) from the ETC. The increased substrate available in the hepatic cells of diabetic rats, especially glucose and fatty acids, may lead to an increase in oxidative phosphorylation which, together with the existence of a reduced function of CIII, may lead to increased $O_{2}^{•-}$ production by increase in the escape of ETC electrons that react with O_2_ (Raza et al., 2011; Blake and Trounce, 2013). Therefore, this might be the key process related to cell lesions caused by hyperglycemia.

We also observed an increase in the activity of antioxidant enzymes SOD and CAT in DM group. The overexpression of SIRT3, also observed in DM group, may have induced an increase in the activity of the SOD enzyme through deacetylation, since SOD is also a target regulated by SIRT3 (Chen et al., 2011). The overproduction of $O_{2}^{•-}$ in the mitochondrial ETC of diabetic rats could contribute to the induction of increased expression of SIRT3 and, consequently, an increase in deacetylation of SOD with a resultant increase in enzyme activity, since Chen et al. (2011) demonstrated that the overproduction of ROS stimulates the expression of SIRT3. SOD is responsible for disrupting the $O_{2}^{•-}$ in hydrogen peroxide (H_2_O_2_); consequently, increased H_2_O_2_ production would be responsible for increased CAT enzyme activity. In oxidative stress, the positive regulation of SOD and CAT activity is not necessarily protective, but rather a compensatory response to eliminate excess $O_{2}^{•-}$ and H_2_O_2_, considering that even increased, the enzyme did not prevent damage to lipids. These data confirm the existence of an increase in the production of ROS and, consequently, an increase in oxidative stress in diabetic rats.

With the restoration of the activity of the ETC complexes after treatment of the diabetic rats with JPE, there was a decrease in both lipid peroxidation and activity of the antioxidant enzymes SOD and CAT. This decreased activity of the enzymes can be explained by a probable lower production of $O_{2}^{•-}$ and, consequently, lower H_2_O_2_ production, which led to a decrease in oxidative damage to lipids. With the lower production of ROS in ETC, the enzymes would be less necessary leading to a decrease in their activity. In addition, down-regulation of SIRT3 in the DM group treated with JPE may also be responsible, at least in part, for the reduction of SOD activity.

As an experimental research, this study has some limitations, such as the rat model and the induced DM model, which makes it difficult to extrapolate the data to humans. In addition, other molecular markers such as SIRT1 as well as ATP, NADH levels, and the activity of antioxidant enzymes such as glutathione peroxidase and glutathione reductase were not investigated, which could have help to explain the observed results better. However, the results obtained in this study are important since this is the first study that showed the modulating effect of JPE on the complexes of mitochondrial ETC and the expression of SIRT3, demonstrating the mechanisms by which JPE could reduce the damage caused by DM. In addition, this study opens the prospect for further studies to deepen the understanding about the mechanisms of action of JPE.

## Conclusion

These findings together demonstrate a novel biological effect for JPE with the potential to regulate the expression of SIRT3 and thereby regulate the function of the mitochondrial ETC and, consequently, reduce oxidative damage in liver of diabetic rats. Thus, the JPE can become an important source of phenolic compounds as a possible adjuvant treatment of DM-related complications. Further studies are needed to completely elucidate this mechanism of action.

## Data Availability Statement

The raw data supporting the conclusions of this article will be made available by the authors, without undue reservation.

## Ethics Statement

The animal study was reviewed and approved by Ethics Committee of the University of Caxias do Sul.

## Author Contributions

MS was the chief investigator of this research, contributed to conceptualization, fundraising, methodology development, and research supervision. MJ contributed to the conceptualization of the research project, fundraising, methodology development, and research supervision. LM, DS, and DG contributed to the collection of samples and data and analysis of them, in addition to contributing to the literature review. CC contributed to the conceptualization of the research, development of the methodology, collection of samples and data and analysis of the same, and the preparation of the original draft of the manuscript. All authors contributed to the review and final preparation of the manuscript.

### Conflict of Interest

The authors declare that the research was conducted in the absence of any commercial or financial relationships that could be construed as a potential conflict of interest.
